# Mapping eating disorders in adolescents and young adults: an investigation of geographic distribution and access to care in Ontario, Canada

**DOI:** 10.1186/s40337-024-01098-6

**Published:** 2024-09-09

**Authors:** Nelson Pang, Jason M. Nagata, Alexander Testa, Kyle T. Ganson

**Affiliations:** 1 Factor-Inwentash Faculty of Social Work, University of Toronto, Toronto, ON Canada; 2 Department of Pediatrics, University of California, 550 16th Street, 4th Floor, Box 0503, San Francisco, CA 94143 USA; 3 Department of Management, Policy and Community Health, University of Texas Health Science Center at Houston, Houston, TX USA

**Keywords:** Geographic information systems, Ontario, Adolescents, Eating disorders, Treatment

## Abstract

**Background:**

There is limited research on the spatial distribution of eating disorders and the proximity to available eating disorder services. Therefore, this study investigates the distribution of eating disorders among adolescents and young adults in Ontario, Canada, with a specific focus on geographic disparities and access to publicly-funded specialized eating disorder services.

**Methods:**

A community sample of 1,377 adolescents and young adults ages 16–30 across Ontario between November and December 2021 participated in this study and completed the Eating Disorder Examination Questionnaire. Utilizing Geographic Information System (GIS) technology, we mapped the geographic prevalence of eating disorders and examined proximity to specialized eating disorder services. Multiple linear and logistic regression analyses were utilized to determine the association between geographic region and eating disorder symptomatology. Additionally, t-tests were utilized to examine differences between time/distance to specialized services and clinical risk for eating disorders.

**Results:**

Applying geospatial analysis techniques, we detected significant spatial clusters denoting higher eating disorder scores in rural areas and areas with fewer specialized services. Likewise, our findings report disparities between rural and urban areas, suggesting that rural regions exhibit elevated rates of eating disorders. There were no associations between distance/time to services and eating disorder symptomology.

**Conclusions:**

The discrepancies in eating disorder symptomology between urban/rural may stem from stigma and unique socio-cultural contexts in rural communities. The study underscores the need for targeted intervention, including telehealth, in addressing the eating disorder challenges faced by adolescents and young adults in rural regions.

## Abstract (plain language summary)

This study explores how common eating disorders are among adolescents and young adults in Ontario, Canada, with a specific focus on the geographic disparities of eating disorders. This study uses mapping technology to assess where eating disorders were more common and how close these areas were to specialized eating disorder treatment services. The findings showed that places with fewer services, especially rural areas, had higher rates of eating disorders. However, there wasn’t a clear link between how far people lived from these services and the severity of their eating disorders. This may suggest that those in rural areas might struggle more with eating disorders due to greater stigma and different social and cultural factors compared to urban areas. This study emphasizes the need for targeted interventions, like telehealth, to address these disparities. This research is pivotal in guiding equitable healthcare solutions for eating disorders, particularly in underserved rural communities.

## Background

Eating disorders, including anorexia nervosa, bulimia nervosa, and binge eating disorder, are a significant public health concern affecting up to 4% of the adolescent and young adult population globally [1]. Eating disorders encompass a range of psychological and physiological complexities, leading to a lower quality of life, debilitating symptoms, and mortality [2]. Research has found that anorexia nervosa has the second highest mortality rate among psychiatric disorders in the DSM-5, second only to opioid use disorder [3].

Eating disorders typically develop between late adolescence and young adulthood, with the average age onset between the ages of 15 to 24 [4]. Adolescence is a developmental period defined by biological growth and major social role transitions, typically between the ages of 10 and 25, whereas young adulthood is a stage that generally follows adolescence and is characterized by the continuation of full physical development and the transition into independent adulthood, typically defined as the ages between 18 and 30 [5]. This age range is critical as it encompasses key developmental periods, including the transition from adolescence to adulthood, which can be associated with various stressors and challenges [5]. During this time, individuals are forming their identities and experiencing significant life changes, all of which can contribute to the development and exacerbation of eating disorders [6].

Despite prior research showing that there are many treatment modalities for eating disorders, only a small proportion of people living with eating disorders receive clinical treatment [7–9]. The lack of treatment utilization among those with eating disorders can be attributed to various factors, including the lack of identification and diagnosis, financial barriers, limited proximity of access to services, and reluctance to seek treatment [4, 6, 9–11]. However, limited research has been conducted to analyze the geographic distribution of eating disorders and the proximity to available eating disorder services as potential factors.

Proximity and travel to health care services have an important role in access to mental health care. Long geographical distances and travel times can be a barrier to health services, leading to underutilization of services and poorer health outcomes [12, 13]. Prior research has documented that mental health service use decreased as travel time increased [14]. Proximity and travel to health care services can be particularly difficult in rural areas where there are fewer services available [15]. Rural-urban differences have been examined for mental health disorders including anxiety, depression, substance use, and suicide with inconsistent findings [16–18].

While the research on rural-urban differences in the prevalence of eating disorders is limited, there are few studies that examine these disparities. A study in the Netherlands found that the incidence of bulimia nervosa was significantly higher in urbanized areas compared to rural areas, with the incidence increasing as urbanization increased; however, there was no significant difference in the incidence of anorexia nervosa between urban and rural areas​ [19]. Similarly, research from Italy has shown that urban adolescents are more likely to exhibit symptoms of eating disorders, particularly bulimia nervosa, compared to their rural counterparts, whereas the prevalence of anorexia nervosa does not significantly differ between urban and rural settings [20]. Conversely, research in the United States suggests that rural adolescents may be at an elevated risk for disordered eating behaviors overall [21]. However, specific data on bulimia nervosa and anorexia nervosa is less detailed but it suggests that self-induced vomiting was higher in rural areas [21]. The heightened risk of disordered eating in rural areas in the United States could be attributed to factors including socioeconomic disadvantage, food insecurity, and limited healthcare access which are more prevalent in rural areas [21]. Unlike the Netherlands and Italy, where urban areas exhibit a clearer association with eating disorders, research from the United States suggests that rural environments might present unique challenges that increase the risk of disordered eating [19–21]. These differences highlight the complex interplay of cultural, social, and environmental factors influencing eating disorder prevalence across various geographical contexts and underscore the need for further research to better understand these patterns.

Furthermore, the distribution and access to mental health services, including specialized care for eating disorders, often vary between rural and urban areas [22, 23]. Rural communities frequently encounter distinct challenges related to the prevalence, diagnosis, and treatment of mental health disorders [21]. Previous research has identified that rural areas may experience higher rates of certain mental health issues, such as suicidality, due to limited access to healthcare, stigma surrounding mental health, and reduced availability of specialized services [24, 25]. Conversely, urban areas generally offer greater access to healthcare services and specialized services [15, 26].

This study aims to contribute to this understanding by [1] investigating the geographic distribution of eating disorders among adolescents and young adults in Ontario and [3] assessing potential rural-urban disparities in access to treatment centers. Understanding the unique challenges faced by adolescents and young adults with eating disorders in Ontario is crucial for developing effective prevention strategies, early intervention programs, and targeted treatment approaches. In Ontario, the health care system is primarily publicly funded, ensuring that all residents can access essential medical services without payment at the point of care. This system, known as the Ontario Health Insurance Plan (OHIP), covers a wide range of health services, including hospital visits, medical appointments, and certain surgical procedures. In Ontario, most publicly funded eating disorder treatment programs are situated within hospital settings, requiring a higher level of eating disorder symptom acuity. Therefore, most outpatient eating disorder treatment (i.e., individual/family therapy) would require payment out-of-pocket or via supplemental health insurance. By examining the geographic distribution of adolescents and young adults affected by eating disorders in Ontario, valuable insights into the spatial patterns and disparities in access to care services across different regions of the province can be gained. This information will enable healthcare providers, policymakers, and stakeholders to identify areas of need and improve the delivery of specialized care to adolescents and young adults with eating disorders. This is an exploratory study given the dearth of research in this area of inquiry in Canada.

## Methods

### Data source

Data was collected from a community sample of 2,731 adolescents and young adults from the Canadian Study of Adolescent Health Behaviors. Participants were recruited using a non-probability sampling method using online recruitment using Instagram and Snapchat advertisements between November and December 2021. Eligibility criteria include being between the ages of 16 and 30 years old, currently living in Canada, and being able to complete the survey in Canada. Survey data was collected and managed using Qualtrics. By completing the survey, participants were entered into a draw to win one of two Apple iPads or one of 20 $25 Starbucks gift cards. The survey took approximately 45 min to complete. A subset of 1,381 participants from Ontario were utilized for this study given this is the most populous province in Canada and has a robust eating disorders prevention and treatment strategy [27]. After linking valid postal codes to geographic data for this study the final analytical sample was 1,377 participants. The study received ethical approval from the research ethics board at the University of Toronto (#41707), and informed consent was obtained from all participants through a checkbox option.

### Measures

#### Eating disorder symptomology

To assess and identify eating disorder symptomology the Eating Disorder Examination Questionnaire (EDE-Q) version 6 was used [28]. The EDE-Q is a self-report questionnaire that examines disordered eating attitudes and behaviours within the past 28 days. The EDE-Q consisted of 28 items and the value for Cronbach’s Alpha for this sample was α = 0.91 indicating good internal consistency. Eating disorder scores are measured with a 7-point (range: 0–6) ordered response. Higher mean scores indicate greater eating-related symptomology. A cut-off of 2.48 was used as a marker of clinical significance [29].

### Geospatial data

To identify and utilize geospatial data participants were asked for their postal code. With the postal code we were able to connect participants to the major public health region they are currently living and the rurality of the region. In Ontario, Canada the geographic area covered by a postal code varies widely, ranging from a few city blocks in urban areas to large rural regions encompassing several towns. The rurality of the regions was determined using the Statistics Canada Peer Groups classification [30]. The Statistics Canada Peer Groups classification categorizes Canadian regions based on socio-economic and demographic factors, such as income, education, employment, housing, and population density. As a result, there are five groups for geographic regions: highest urban, mainly urban, sparsely urban-rural mix, and mainly rural [30]. The “highest urban” group includes regions with the highest population densities, extensive urban infrastructure, and diverse economic activities, such as Toronto and Ottawa. The “mainly urban” group features smaller cities and large towns with significant urban infrastructure and services, such as London and Kitchener-Waterloo. The “sparsely urban-rural mix” group consists of regions with a combination of small urban centers and rural areas, characterized by lower population densities and a blend of urban and rural economic activities, such as the Thunder Bay District. Lastly, the “mainly rural” group encompasses areas with low population densities, predominantly rural landscapes, and economies based on primary industries like agriculture and resource extraction, such as Huron County. By grouping regions into categories that reflect different levels of urbanization, the system provides a robust proxy for rurality, allowing for meaningful comparisons and analyses in informed policymaking and research in Canada [30].

#### Eating disorder services

To identify eating disorder services, the research team searched through the National Eating Disorder Information Centre (NEDIC) website for resources in Ontario. NEDIC is a charitable Canadian organization dedicated to providing information, resources, and support to individuals affected by eating disorders. Eating disorder providers that were covered by provincial health insurance focused on adolescents and young adults were searched for. Specifically, treatment was searched for anorexia nervosa, binge eating disorder, bulimia nervosa, avoidant and restrictive food intake disorder, disordered eating, and any other unspecified feeding or eating disorder. Services identified spanned different levels of care, including both inpatient and outpatient care. The addresses of these services were the primary data point and were combined with participants’ geospatial data to identify proximity to services. A total of 53 public services specializing in eating disorder services in Ontario were identified and we were able to map 48 services to geospatial data due to data formatting issues and/or incorrect data.

#### Sociodemographic variables

Sociodemographic variables included race/ethnicity, gender (cisgender woman, cisgender man, transgender and gender expansive), sexual orientation, personal income, and highest level of education completed.

### Analysis

The analysis involved the creation of maps to explore geographic clusters of eating disorders and generate maps of high and low eating disorder scores. To analyze gaps in care, we examined where eating disorder services are located in Ontario relative to EDE-Q scores (i.e., severity levels of eating disorder symptomology). To examine the coverage of care and services distance and drive time will be used as a proxy for access to services. A buffer analysis was conducted to create zones around public healthcare centers specializing in eating disorder care using distance and drive time. Statistical analysis was conducted to examine differences in EDE-Q global scores based on location, rurality, and access to services. Spatial analysis was conducted within the ArcGIS software to explore areas with higher eating disorder scores among adolescents and young adults. To examine proximity to care drive time and drive distance buffers were created within the ArcGIS software. A 30-, 45- and 60-minute drive time buffer and a 5-, 25-, 55- kilometre driving distance buffer was created for public healthcare centers that specialize in eating disorder care. This approach of creating buffers allows us to analyze the accessibility of these centers based on both time and distance.

Two-sample t*-*tests were used to determine whether there were significant differences between EDE-Q clinical cut-off by participant travel time and distance to service providers. One-way analyses of variance (ANOVAs) were used to determine whether there were significant differences in EDE-Q global scores by public health region and geographic regions. Chi-square tests for independence were used to determine whether there was a significant association between public health regions, rurality, and clinical eating disorder risk. Adjusted analyses were conducted using linear and logistic regressions to determine the associations between region and eating disorder symptomology, while adjusting for the sociodemographic variables. Statistical significance was determined using two-sided *p* < 0.05. Geospatial analysis was conducted using ArcGIS and statistical analysis was conducted using R Statistical Software. Descriptive statistics using means, standard deviations, and frequencies were used to describe the sample.

## Results

The majority of participants identified as cisgender women (53.2%), followed by cisgender men (40.5%) (See Table 1 for full demographics). The majority of participants (58.6%) identified as White, followed by East Asian (11.2%), and multi-racial (10.3%). The average age of participants was 23.0 (SD = 3.93). In addition, 56.6% of participants had completed a college degree or more. Participants were evenly distributed across the regions of Ontario except for the Northern region. The majority of participants lived in highly (47.4%) and mainly urban settings (33.1%). The average EDE-Q global score was 2.2 (SD = 1.5), and 37.3% of participants scored 2.48 or higher, indicating a clinical risk of eating disorders.

**Table 1:** Demographic characteristics of participants in ontario from the canadian study of adolescent health behaviors (*N* = 1,377)

Gender	*n* (%)
Cisgender woman	732 (53.2%)
Cisgender man	558 (40.5%)
Transgender and gender expansive	82 (6.0%)
Sexual orientation	
Heterosexual	809 (58.8%)
Bisexual	248 (18.0%)
Queer, questioning, other	203 (14.7%)
Gay/lesbian	116 (8.4%)
Race/ethnicity	
White	807 (58.6%)
East Asian	154 (11.2%)
Multi-Racial	142 (10.3%)
South Asian	122 (8.9%)
Black	56 (4.1%)
Middle Eastern	39 (2.8%)
Latino	29 (2.1%)
Other	19 (1.4%)
Indigenous	9 (0.7%)
Highest completed education	
High school or less	583 (42.3%)
College or undergraduate degree	586 (42.6%)
Master’s degree or higher	193 (14.0%)
Other	14 (1.0%)
Annual personal income	
$24,999 or less	777 (56.4%)
$25,000-$49,999	216 (15.7%)
$50,000-$74,999	226 (16.4%)
$75,000-$99,999	89 (6.5%)
$100,000 or more	63 (4.6%)
Public health region	
Toronto	348 (25.3%)
Central	334 (24.3%)
East	324 (23.5%)
West	322 (23.4%)
North	49 (3.6%)
Geographic region^a^	
Highest urban	653 (47.4%)
Mainly urban	456 (33.1%)
Sparsely urban-rural mix	159 (11.5%)
Mainly rural	109 (7.9%)
Age	
Mean (SD)	23.0 (3.93)
Median [Min, Max]	23 [15,30]
EDE-Q global score	
Mean (SD)	2.24 (1.50)
Median [Min, Max]	1.88 [0, 5.95]
At clinical risk for eating disorder	513 (37.3%)

*Note*^a^Urban/Rural was determined using Statistics Canada Peer Groups classification

M = Mean; SD = Standard deviation; EDE-Q = Eating Disorder Examination Questionnaire

**Fig. 1:**
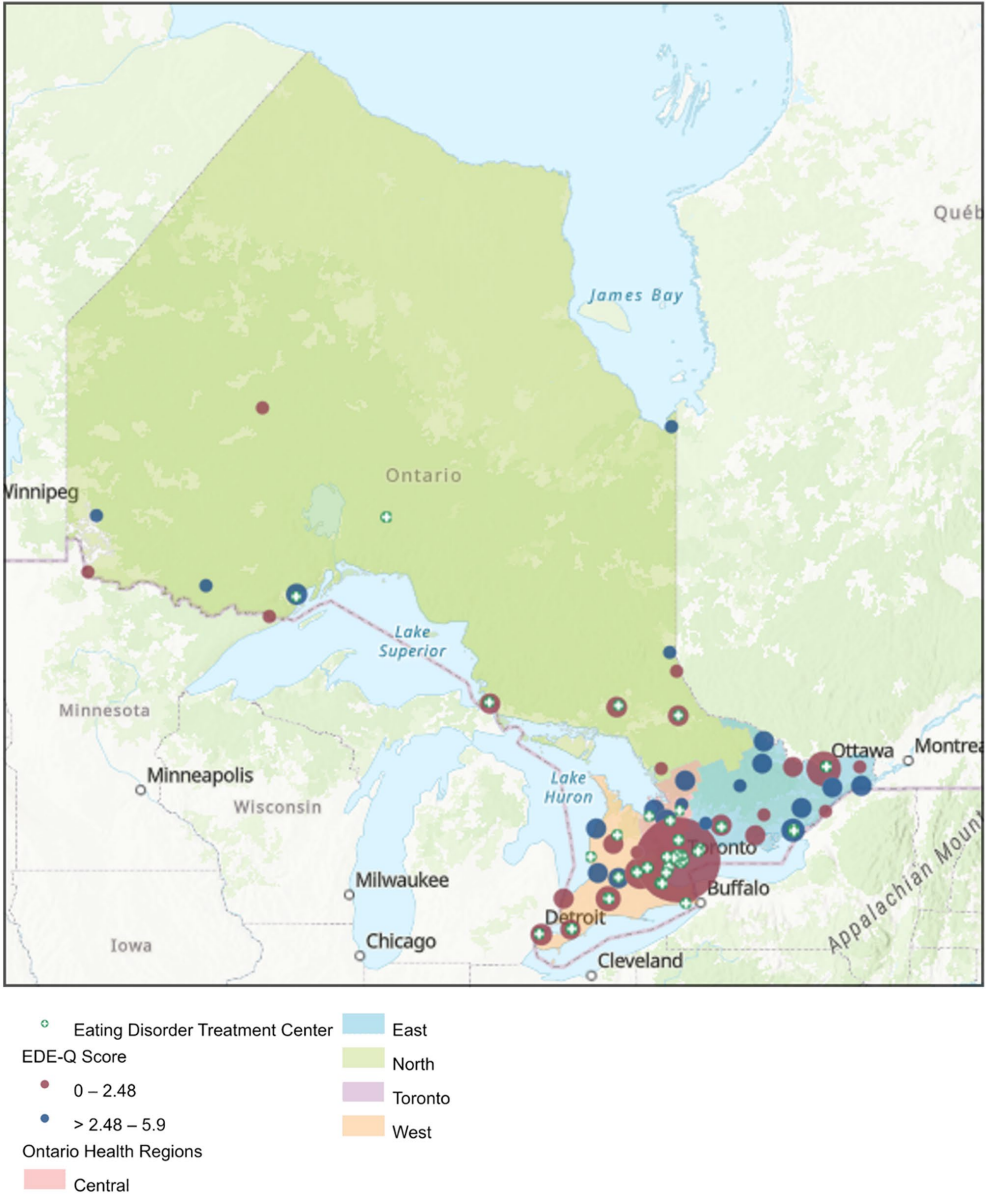
Specialized public eating disorder services in ontario and clusters of eating disorders scores in ontario

The mapping of EDE-Q scores revealed spatial clusters of areas of clinical risk for eating disorders (see Fig. 1). This map shows the clustering of higher eating disorder scores appears to be in more rural areas and less in large city centers like Toronto. The majority (54.2%) of services are focused in urban areas (See Fig. 1; Table 2).

**Table 2:** Public health region and geographic area of specialized public eating disorder services in ontario (*n* = 48)

**Major Public Health Region** ^a^	
West	16 (33.3%)
Central	12 (25.0%)
East	10 (20.8%)
North	5 (10.4%)
Toronto	5 (10.4%)
**Urban/Rural** ^b^	
Sparsely urban-rural mix	14 (29.2%)
Mainly urban	13 (27.1%)
Highest urban	13 (27.1%)
Mainly rural	8 (16.7%)

*Note*^a^Public Health Region were determined based on the postal code of the service provider

^b^Urban/Rural was determined using Statistics Canada Peer Groups classification

**Fig. 2:**
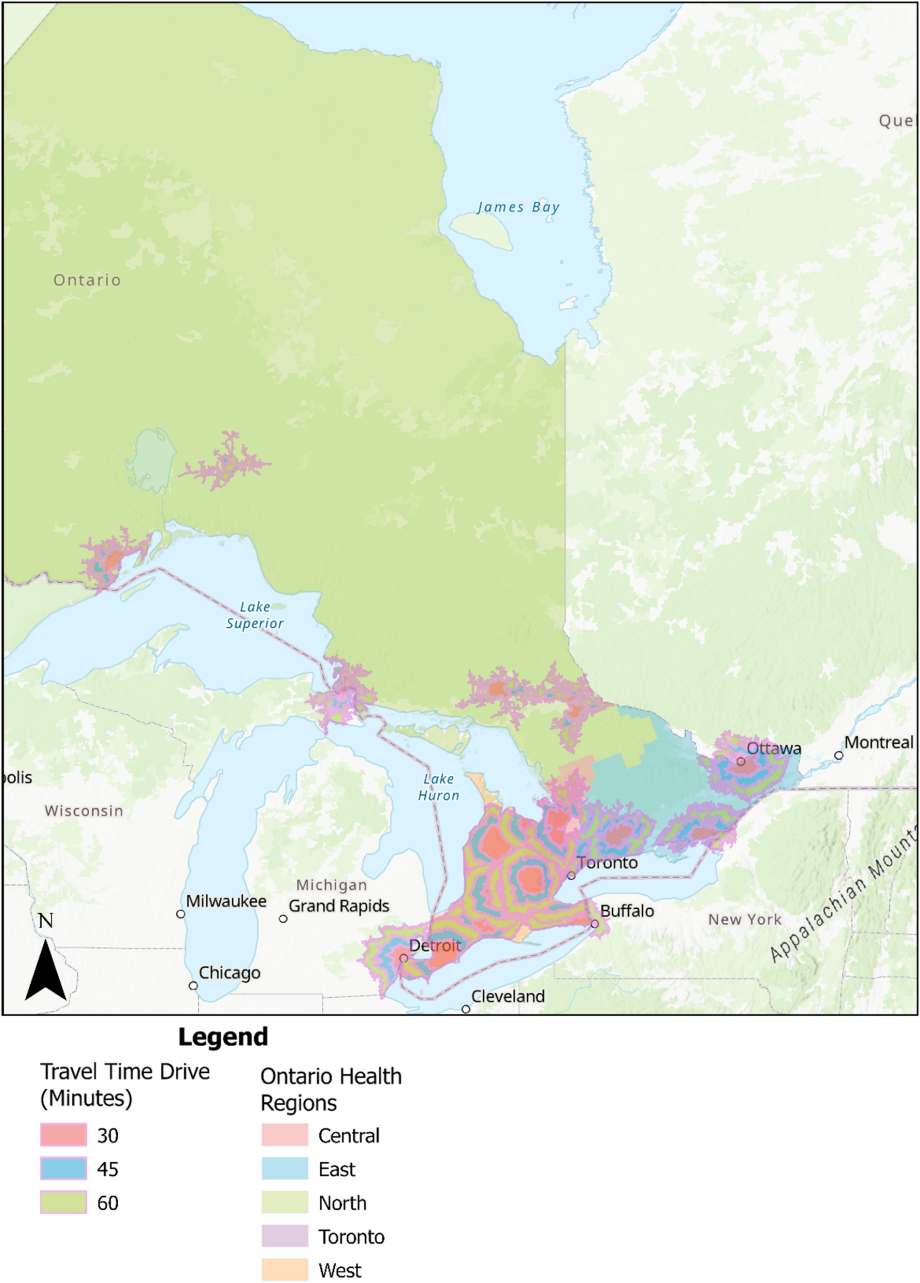
Drive time buffer for specialized public eating disorder services

**Fig. 3:**
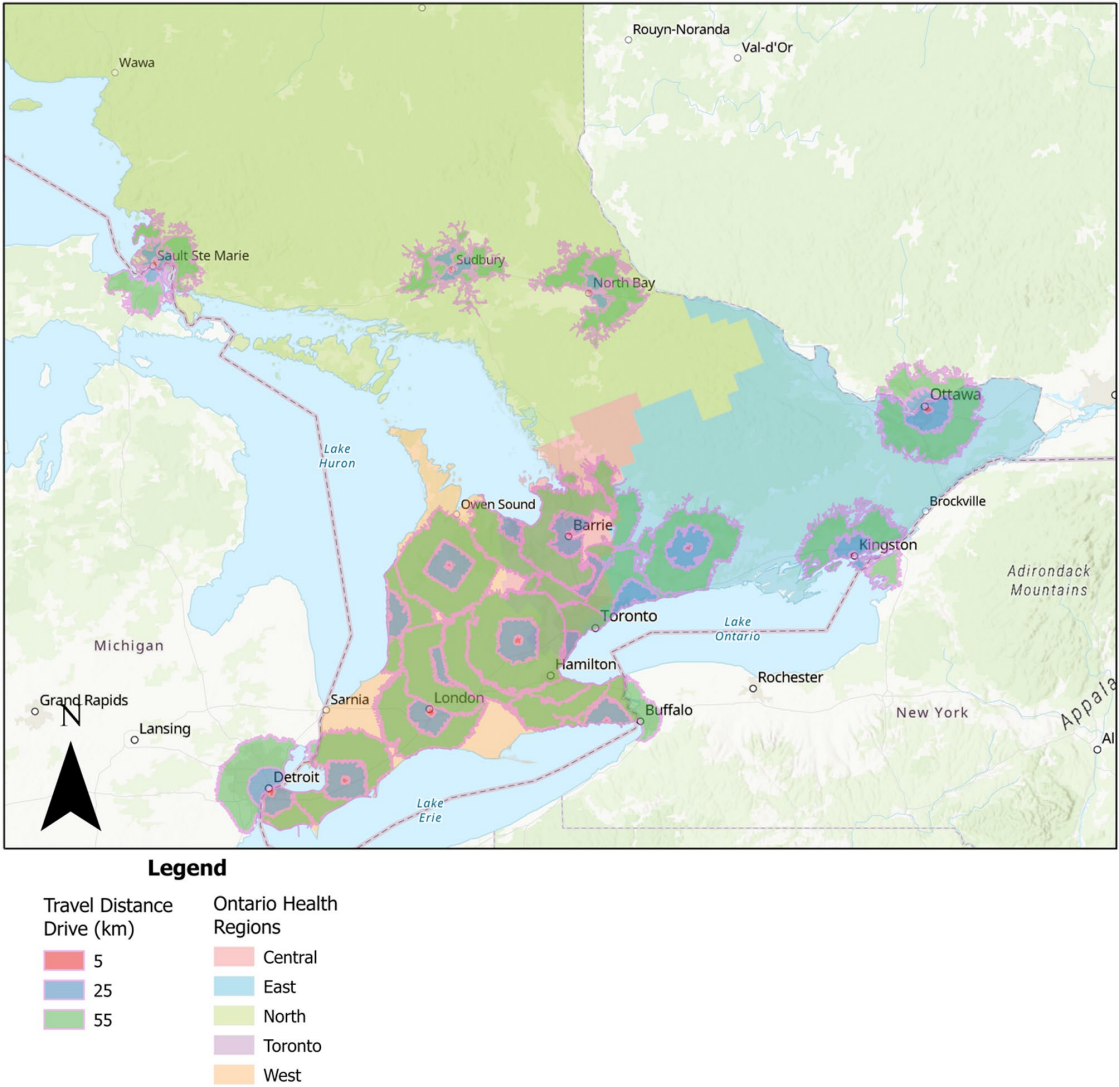
Drive distance buffer for specialized public eating disorder services

Proximity to care was analyzed by examining a 30-, 45- and 60-minute drive time buffer and a 5, 25-, 55 km driving distance buffer to public healthcare centers that specialize in eating disorder care (see Figs. 2 and 3). Based on visual inspections of the maps, public eating disorder services are accessible for most of southern Ontario (i.e., the most populated and urban part of Ontario) but there are fewer services available outside of this area. Overall, most of the services are available and cluster in larger cities (i.e., Toronto).

**Fig. 4:**
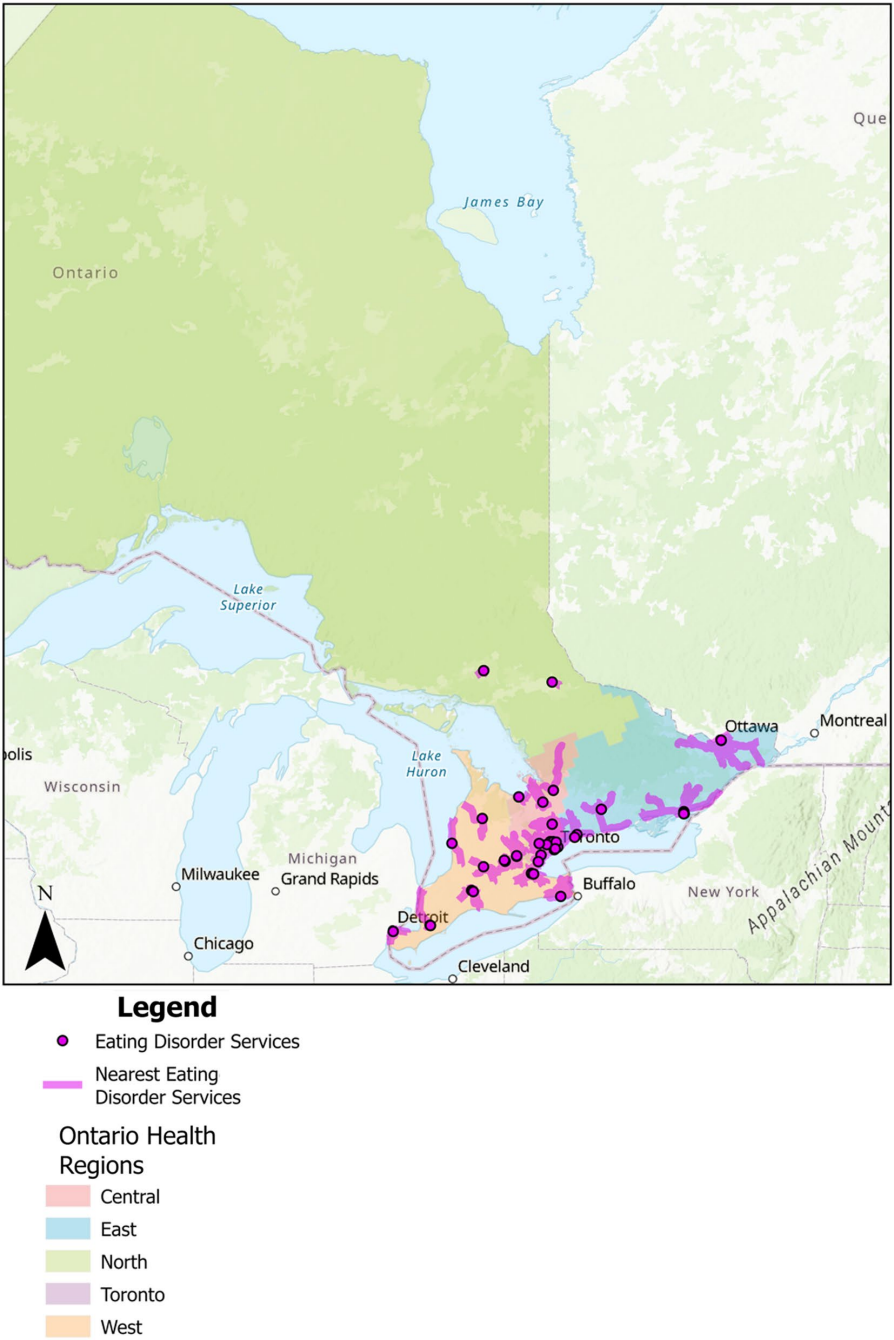
Nearest specialized public eating disorder services

To further examine proximity to care, t-tests were conducted to examine the nearest eating disorder service to participants and clinical risk of eating disorders (See Fig. 4). The average driving distance to access eating disorder services was 9.9 km (SD = 12.6) and driving time was 14.5 min (SD = 11.5). Our t-test results showed no significant differences between driving distance/time to eating disorder services and clinical risk of eating disorders. A two-sample t-test showed no significant difference in total kilometers to eating disorder services between individuals with no clinical risk of eating disorders (M = 9.63) and those with a clinical risk of eating disorders (M = 10.03), 𝑡 (1088.7) = − 0.561, 𝑝 = 0.575. Similarly, a two-sample t-test showed no significant difference in total minutes needed to travel to eating disorder services between individuals with no clinical risk of eating disorders (M = 14.22) and those with a clinical risk of eating disorders (M = 14.55), 𝑡 (1085.4) = − 0.523, 𝑝 = 0.601.

**Fig. 5:**
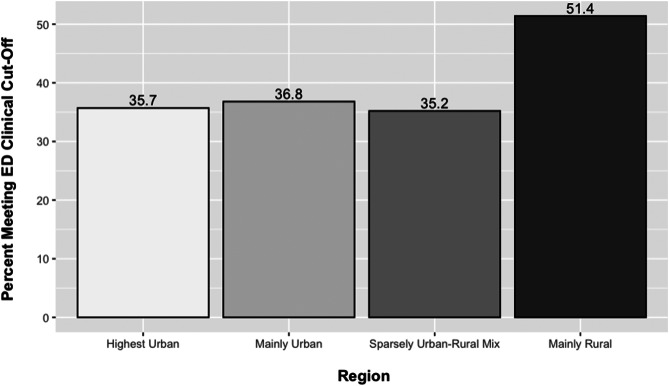
Percent of participants meeting eating disorder clinical cut-off by geographic region. *Note* Significant differences (*p* = 0.0183) between regions among those at clinical risk for eating disorders

ANOVA results revealed that there were significant differences in EDE-Q global scores between public health regions (F (4,1310) = 4.159, *p* = 0.002) and geographic regions (F (3,1311) = 5.03, *p* = 0.002). Specifically, EDE-Q global scores were highest in the public health unit Central (mean = 2.5 [SD = 1.5]) and lowest in North (mean = 2.0 [SD = 1.5]) (See Table 3). For geographic regions, EDE-Q global scores were highest in mainly rural areas (mean = 2.8 [SD = 1.6]) and lowest in the highest urban areas (mean = 2.2 [SD = 1.4]) (See Table 3). Likewise, there were significant associations between clinical risk for eating disorders and public health units and geographic regions (See Fig. 5). For example, 53.3% of participants living in a mainly rural region were at clinical risk for an eating disorder. A chi-squared test revealed a significant association between geographic regions and clinical risk of eating disorders, 𝜒^2^ (3, 𝑁 = 1374) = 10.024, 𝑝 = 0.018 χ^2^ (3, *N* = 1374) = 10.024, *p* = 0.018. This suggests that the distribution of eating disorder risk varies significantly across different levels of urbanization. Likewise, a chi-squared test revealed a significant association between public health region and eating disorder risk, 𝜒^2^ (4, 𝑁 = 1374) = 20.245, 𝑝 < 0.001 χ^2^ (4, *N* = 1374) = 20.245, *p* < 0.001. This indicates that the distribution of eating disorder risk varies significantly across different regions.

Adjusted regression analyses revealed that public health units and geographic regions were significantly associated with eating disorder symptomology (Table 4). Specifically, those in mainly rural areas had significantly higher odds (OR: 1.81, 95% CI [1.09,3.01], *p* = 0.021) of being at clinical risk for an eating disorder compared to those in the highest urban areas. Likewise, those in the Central (OR: 2.06, 95% CI [1.40, 3.06], *p* < 0.001) public health region had significantly higher odds of being at clinical risk for an eating disorder compared to those in the Toronto region.

**Table 3:** ANOVA and chi-square of eating disorders symptomology by geographic area

Public Health Region								
	Central (*N* = 334)	East (*N* = 324)	North (*N* = 49)	Toronto (*N* = 348)	West (*N* = 322)	Total Sample (*N* = 1377)	P	Effect Size ^a^
**EDE-Q Global**								
Mean (SD)	2.45 (1.50)	2.35 (1.53)	2.01 (1.48)	2.02 (1.38)	2.19 (1.54)	2.24 (1.50)	0.002	0.012
**ED Clinical Cut-Off**								
Yes	149 (44.6%)	130 (40.1%)	13 (26.5%)	106 (30.5%)	115 (35.7%)	513 (37.3%)	< 0.001	0.124
**Geographic Region**								
	Highest urban (*N* = 653)	Mainly urban (*N* = 456)	Sparsely urban-rural mix (*N* = 159)	Mainly rural (*N* = 109)	Total Sample (*N* = 1377)			
**EDE-Q Global**								
Mean (SD)	2.18 (1.44)	2.22 (1.51)	2.19 (1.53)	2.78 (1.65)	2.24 (1.50)		0.002	0.011
**ED Clinical Cut Off**								
Yes	233 (35.7%)	168 (36.8%)	56 (35.2%)	56 (51.4%)	513 (37.3%)		0.018	0.087

^a^ Determined using η^2^ for ANOVA & Cramer’s V for chi-square tests

**Table 4:** Linear and logistic regressions betweenn geographic region and eating disorder symptomology

	EDE-Q Global Score			EDE-Q Clinical Risk (EDE-Q > 2.48)	
	B (95% CI)	p	R^2^	OR (95% CI)	p
**Geographic Region**			0.175		
Intercept	**1.34 (0.21**,** 2.47)**	**0.021**		0.23 (0.04, 1.46)	0.121
Ref: Highest Urban					
Mainly rural	**0.52 (0.20**,** 0.84)**	**0.001**		**1.81 (1.09**,** 3.01)**	**0.021**
Mainly Urban	-0.02 (-0.21, 0.17)	0.843		0.96 (0.71, 1.30)	0.795
Sparsely urban-rural Mix	0.07 (-0.21, 0.34)	0.629		0.99 (0.64, 1.55)	0.992
**Public Health Unit**			0.177		
Intercept	0.96 (-0.19,2.10)	0.102		0.12 (0.02, 0.82)	0.030
Ref: Toronto					
Central	**0.46 (0.22**,** 0.70)**	**< 0.001**		**2.06 (1.40**,** 3.06)**	**< 0.001**
East	**0.27 (0.04**,** 0.51)**	**0.024**		1.39 (0.95, 2.05)	0.091
North	0.21 (-0.24,0.68)	0.362		0.94 (0.42, 2.01)	0.874
West	0.20 (-0.04, 0.45)	0.100		1.34 (0.91, 2.00)	0.142

*Note* Each column represented the abbreviated outputs of 2 regression models with geographic region and Public Health as the independent variables and EDE-Q global score and EDE-Q Clinical Risk as the dependent variables

Values in **bold** are significant with *p* < 0.05

^a^ Analyses adjusted for age, race/ethnicity, gender, sexual identity, income, and highest level of education completed

EDE-Q = Eating Disorder Examination Questionnaire; B = Coefficient from linear regression; OR = Odds Ratio from logistic regression; CI = Confidence interval

## Discussion

The findings of this study shed light on a significant and previously understudied aspect of eating disorders among adolescents and young adults in Ontario and the disparity in prevalence across geographic regions of the province. Our analyses indicate that rural areas exhibited higher severity of eating disorders symptomology, which aligns with emerging evidence suggesting that rural adolescents may be at increased risk for eating disorders [20, 21]. The higher severity of eating disorder symptoms in rural regions could be attributed to fewer healthcare options. In particular, limited access to specialized services for eating disorder treatment may lead to delayed diagnosis and intervention [31]. However, it is important to note that this study did not measure service utilization directly or ask participants about their ability to access service providers, which should be acknowledged as a limitation.

While our statistical analysis did not find a significant difference between distance or travel time to accessing services and eating disorder risk, our visual inspection of the maps revealed a notable disparity in the availability of specialized services in rural regions compared to urban areas. This observation, although not statistically significant, is supported by the broader context of healthcare access challenges commonly faced by rural communities. Visual inspection can provide an additional layer of understanding when interpreting spatial data, particularly in identifying geographical trends and clusters that may not be apparent through statistical methods alone [32]. Additionally, telehealth services are available to people living in rural areas in Canada, and many eating disorder programs in Ontario provide virtual care. Therefore, it is possible that even those in rural areas are able to access care when needed despite travel distance and time. Thus, other factors may play a more significant role in these regions.

In rural areas, adolescents and young adults may spend increased time on screens for socialization, leading to heightened exposure to social media and prevalent body ideals, which can potentially lead to the onset of eating disorders [33]. The potential isolation of rural communities can foster feelings of loneliness and a diminished sense of social support, both of which have been identified as risk factors for mental health challenges, including eating disorders [34]. Economic challenges, such as unemployment or financial instability, are prevalent in many rural areas, which further exacerbate the risks of mental health issues and eating disorders [35, 36]. Rural regions in Ontario are often socioeconomically disadvantaged compared to more urban regions [37], which may be associated with a higher prevalence of eating disorders [38, 39]. Likewise, food insecurity is also more common among rural areas compared to urban areas [40, 41], which has also been found to be associated with eating disorders [35]. Furthermore, the socio-cultural context of rural areas may contribute to the differences in eating disorder symptomology. For example, previous research has found that rural adolescents and young adults have unique experiences with body image and weight stigma [21].

Prior research has found that rural populations have poorer health outcomes and are more likely to have limited healthcare access for numerous reasons, including distance to services, lack of services, and stigma that decreases healthcare utilization [42–44]. Our finding are consistent with research on the rural-urban divide on health care access generally but expands this research to exploring proximity to eating disorder treatment centers, which has gone overlooked previously [15, 45]. The stigma surrounding mental health in rural communities might also hinder individuals from seeking help, thereby exacerbating the prevalence of untreated eating disorders [25, 46]. Furthermore, longer waitlists and limited provider availability are often more prevalent in rural areas where there are fewer treatment centers [47]. This can lead to delays in receiving care, prolonged periods of untreated symptoms, and increased severity of the disorder by the time treatment is accessed [48]. Additionally, the lower density of treatment centers paired with a higher density of eating disorder cases can strain existing resources, leading to reduced quality of care and less personalized treatment options.

While this study unveils disparities in eating disorder symptomology between areas of different rurality, further research is needed to understand the multifaceted cause of this disparity. However, our study was able to identify regions with elevated eating disorder prevalence and clusters of cases, directing attention to areas where intervention efforts are most needed. These findings are crucial for policymakers and healthcare providers to strategize resource allocation and tailor interventions to the specific needs of affected adolescents and young adults in diverse geographical contexts. Our research suggests a need for more specialized eating disorder services in certain areas of Ontario, specifically in more rural regions. Consistent with the Canadian Eating Disorders Strategy clinical guides and training on best practices via telehealth for rural and remote areas are recommended [49]. Although some treatment centers may already provide telehealth services, our study did not specifically identify which centers offer these services. Future research could benefit from mapping the availability of telehealth services to determine gaps and areas for expansion. Ensuring that telehealth services are effectively integrated and accessible can help address the unique challenges faced by rural communities. Efforts to address the higher rates of eating disorders in rural regions must consider the unique challenges and characteristics of these areas, cultural differences, and stigma are essential. More specialized eating disorder services, telehealth services, and community-based outreach programs could play a pivotal role in providing much-needed support to individuals in remote locations [50]. Educating healthcare providers in rural areas about the risk factors and early symptoms of eating disorders, and offering resources for appropriate referrals, can also contribute to linking adolescents and young adults to care to reduce the disparity.

### Strengths and limitations

To our knowledge, this is the first study to use GIS to investigate eating disorders in Canada. By utilizing spatial analysis techniques, we were able to map the distribution of eating disorders and assess their proximity to specialized services, providing a comprehensive overview of the accessibility of care across the province. This information is crucial for identifying regions that require targeted interventions and allocation of resources. However, there are several limitations that should be acknowledged when interpreting the result of this study. Firstly, this study relies on cross-sectional data may limit our ability to establish causal relationships or assess temporal changes in eating disorder prevalence. Understanding temporal changes can provide insights into the evolution of eating disorder patterns and access to care over time. Specifically, exploring temporal changes could reveal trends in the incidence and management of eating disorders over time, highlighting how these patterns may have shifted due to changes in healthcare policies, societal attitudes, or economic factors. Second, the data was collected using a non-probability sampling method from social media users who may not be representative of the larger population and might be more prone to eating disorders, which may impact the external validity of the findings. Furthermore, eating disorder symptomology was measured using a self-report measure rather than a clinical interview, which may impact the accuracy and reliability of the findings. Similarly, this study did not measure service utilization directly or ask participants about their ability to access service providers, which limits our ability to understand access to services. Additionally, given the large age range [16–30], although age was controlled for in the regression models, the small sample size of participants under 18 years old (*n* = 68) prevented in-depth examination of age-related differences in access to treatment. Likewise, given that 42.6% of the sample completed a college degree and 14% completed a master’s degree or higher, it is likely that the sample includes a significant proportion of adults. Adults generally self-enroll in treatment, influenced by work schedules, financial resources, and personal motivation. In contrast, adolescents and young adults depend on parents or guardians to recognize symptoms, make appointments, and provide transportation. These dynamics highlight the need for tailored strategies to improve access to care for both populations. Future research should explore how treatment-seeking behaviors and access to care differ across age groups, particularly the role of parents in facilitating care for minors. Lastly, we are unable to identify the potential socio-cultural factors that influence the disparities of eating disorders between rural and urban contexts.

## Conclusion

In conclusion, this study provides a comprehensive exploration of the prevalence and distribution of eating disorders among adolescents and young adults in Ontario, with a particular emphasis on rural-urban disparities. By utilizing GIS technology and spatial analysis, we illustrate insights into the geographic patterns of eating disorders and their access to specialized care services. The identification of higher prevalence rates in rural regions underscores the urgent need for tailored interventions and equitable resource allocation.

## Data Availability

Data may be made available upon reasonable request.

## Abbreviations

EDE-Q: Eating Disorder Examination Questionnaire
GIS: Geographic Information System
NEDIC: National Eating Disorder Information Centre
OHIP: Ontario Health Insurance Plan
